# LINAC-based stereotactic radiosurgery targeting the hippocampus in mesial temporal lobe epilepsy: a single-center case series

**DOI:** 10.1186/s41016-026-00446-6

**Published:** 2026-09-08

**Authors:** Víctor Alberto García-García, Marco Vinicio Ramírez-Sánchez, Roberto Carlos García-Romero, Isaac Jasso-García, David Garcia-Martinez, Aldo Tavares-Ortega, Andrea Miriam Gonzalez-Gonzalez, Rubí Carolina Estrada-Hernández, María Fernanda Sánchez-Miranda, Guadalupe Castillo-Cardiel, Gabino Cervantes-Guevara, Enrique Cervantes-Pérez, Sol Ramírez-Ochoa, Alejandro González-Ojeda, Clotilde Fuentes-Orozco

**Affiliations:** 1 Departamento de Neurocirugía, Hospital de Especialidades, Centro Médico Nacional de Occidente, Instituto Mexicano del Seguro Social, Guadalajara, Jalisco 44329 México; 2 Centro Médico Nacional de Occidente (CMNO), Unidad de Investigación Biomédica 02, UMAE, Hospital de Especialidades, Instituto Mexicano del Seguro Social (IMSS), Belisario Domínguez #1000, Colonia Belisario Domínguez, Guadalajara, Jalisco 44329 México; 3 Departamento de Bienestar y Desarrollo Sustentable, Centro Universitario del Norte, Universidad de Guadalajara, Colotlán, Jalisco 46200 México; 4 Departamento de Medicina Interna, Hospital Civil de Guadalajara Fray Antonio Alcalde, Guadalajara, Jalisco 44200 México; 5 Facultad de Medicina, Universidad de Colima, Colima, 28040 México

**Keywords:** Drug-resistant epilepsy, Mesial temporal lobe epilepsy, Stereotactic radiosurgery, Linear accelerator, Seizure

## Abstract

**Background:**

Epilepsy is a neurological disorder affecting over 50 million people worldwide, with a global incidence of 7.6 per 1,000 individuals. Mesial temporal lobe epilepsy (MTLE) is characterized by recurrent, unprovoked seizures originating in the temporal lobe. The standard surgical treatment is anterior temporal lobectomy; however, many patients are not suitable candidates due to the risk of adverse effects, including significant memory impairment, language deficits, bilateral temporal involvement, or reluctance to undergo resective surgery. This study aimed to evaluate the anticonvulsant effect of linear accelerator (LINAC)-based radiosurgery in patients with drug-resistant unilateral MTLE.

**Methods:**

A prospective, single-center, exploratory pilot case series was conducted from March 1, 2017, to March 1, 2020, at the Hospital de Especialidades, Centro Médico Nacional de Occidente. Patients aged 18–65 years with a confirmed diagnosis of drug-resistant unilateral MTLE were enrolled. The primary outcome was defined as the achievement of complete freedom from disabling seizures (Engel Class I and ILAE Class 1) at 24 months post-radiosurgery. Secondary efficacy outcomes included achieving a ≥ 50% reduction in seizure frequency. The prescribed dose of 22 Gy was normalized to the 50% isodose line at the isocenter, ensuring ≥ 90% target coverage (V100 ≥ 90%) and a dose gradient < 10%, targeting the hippocampal head and anterior body.

**Results:**

Seven patients were included. Median seizure frequency decreased from 12 seizures/month at baseline to 3.5 seizures/month at 24 months, corresponding to a median reduction of 75.6%.Longitudinal analysis demonstrated a significant reduction in seizure frequency over time (Friedman χ^2^ = 14.62, p = 0.006), with significant differences between baseline and all follow-up periods (p = 0.018). No patient achieved seizure freedom. Kaplan–Meier analysis demonstrated that 71.4% of patients maintained a clinically meaningful seizure reduction (≥ 50% from baseline) throughout the 24-month follow-up. Patients with preoperative auras exhibited a median aura reduction of 60%, whereas de novo auras developed in three previously aura-free patients. Adverse events included visual field deficits in three patients (42.8%), psychiatric complications in two (28.6%), and clinically significant memory decline in one patient with pre-existing cognitive impairment.

**Conclusions:**

LINAC-based stereotactic radiosurgery targeting the hippocampal head and anterior body achieved a clinically meaningful reduction in seizure frequency in selected patients with drug-resistant MTLE. However, complete seizure freedom was not achieved, and delayed adverse events were observed.

**Trial registration:**

The study was approved by the ethics committee of the Hospital de Especialidades, Centro Médico Nacional de Occidente (approval number R-2017–1301-57).

**Supplementary Information:**

The online version contains supplementary material available at https://doi.org/10.1186/s41016-026-00446-6.

## Background

Epilepsy is a neurological disorder that affects more than 50 million people worldwide, with a prevalence of 6.38 per 1,000 inhabitants and 5 million new cases each year. The overall global prevalence of epilepsy has been reported as 7.60 per 1,000 inhabitants [1]. Its etiology varies throughout life; in childhood, the most common causes include genetic and perinatal factors, as well as malformations, whereas in adults, genetic predisposition, acquired brain injuries, infections, and neuronal degeneration are more prevalent. In Mexico, the prevalence is estimated between 3.9 and 41 cases per 1,000 inhabitants [2]. Among focal epilepsies, temporal lobe epilepsy is the most common form of focal epilepsy in adults, accounting for 60% of cases [3]. Mesial temporal lobe epilepsy (MTLE) is characterized by recurrent, unprovoked, and unpredictable seizures that originate in the temporal lobe [4, 5].

Hippocampal sclerosis (HS) is one of the most common histopathological findings, accompanied by loss of neurons in specific regions of the hippocampus. A high percentage of patients with MTLE associated with HS develop drug-resistant epilepsy; therefore, surgical resection represents the main treatment option, with seizure-free rates ranging from 60 to 80% [5, 6]. Surgical approaches for temporal lobectomy include several technical variations, such as standard anterior temporal lobectomy (ATL), anteromedial temporal lobectomy, and selective amygdalohippocampectomy.

The standard technique for treating mesial temporal lobe epilepsy is ATL; however, it is associated with potential adverse effects, including cognitive dysfunction, visual field defects (mainly quadrantanopia), and intracranial haemorrhage [7]. Moreover, significant memory impairment, particularly affecting recent memory, as well as language disturbances, ranging from dysarthria to aphasia (when the dominant hemisphere is involved), represent clinically relevant complications [5].

Due to this risk profile, the presence of bitemporal disease, or patient reluctance to undergo surgical resection, a considerable proportion of patients are not ideal candidates for ATL or other mesial temporal or neocortical resections, despite the potential improvement or even elimination of seizures [8]. In this context, minimally invasive therapies including laser interstitial thermal therapy (LITT), radiofrequency ablation (RFA), and stereotactic radiosurgery (SRS) have emerged as promising alternatives to conventional epilepsy surgery [7].

Radiosurgery is now recognised as a viable option for patients with intractable epilepsy as an alternative to conventional surgery. In cases of mesial temporal lobe epilepsy associated with hippocampal sclerosis, radiosurgery has demonstrated effectiveness comparable to ATL [9]. Our study aims to evaluate the anticonvulsant effect of linear accelerator (LINAC) radiosurgery in patients with MTLE.

## Methods

### Study design

A prospective, single-center, exploratory pilot case series was conducted from March 1, 2017, to March 1, 2020, in collaboration with the Departments of Neurology, Neurosurgery, and Radiation Oncology at the Hospital de Especialidades, Centro Médico Nacional de Occidente (Guadalajara, Jalisco, Mexico), a tertiary referral center.

### Patient selection

Patients aged 18–65 years with a confirmed diagnosis of drug-resistant unilateral MTLE were enrolled. Drug resistance was defined as the failure to achieve seizure control despite the appropriate administration of at least two antiepileptic drugs (AEDs). Eligibility required a baseline frequency of two or more disabling seizures per month, documented prospectively via seizure diaries for 12 months prior to enrollment, along with three serial EEGs and MRI findings consistent with the diagnosis.

Patients were excluded if their epileptogenic foci was extra-mesial, multifocal, or bilateral, or if MRI findings were inconsistent with MTLE. Additionally, patients were excluded if they failed to attend follow-up visits for more than one month, did not participate in quarterly evaluations, or voluntarily withdrew from the study protocol.

Patients were offered both ATL and LINAC as therapeutic alternatives. Although ATL remains the conventional standard approach, LINAC was selected in these cases after informed shared decision-making, mainly due to patient preference for a less invasive treatment, refusal of open craniotomy. During the study period, LINAC-based surgery was the only radiosurgical platform available at our institution; therefore, alternative radiosurgical modalities such as Gamma Knife and Cyberknife could not be offered.

The pre-therapeutic standardized diagnostic protocol integrated clinical semiology and 12-month prospective seizure monitoring via patient diaries. Patient signs and symptoms were evaluated by the Departments of Neurology and Neurosurgery, with a specific focus on identifying mesial temporal symptomatology. The number of days with seizures and auras during the preceding 12 months was quantified using outpatient seizure diaries.

### Serial EEG

Patients underwent scalp EEG at the Department of Neurology utilizing the international 10–20 system for electrode placement (recording a minimum of eight channels, including referential, longitudinal bipolar, and transverse bipolar montages). The acquired recordings were evaluated in conjunction with the Neurology service to determine the epileptogenic foci and seizure propagation patterns. Three 30-min EEGs were performed at one- to two-day intervals prior to treatment.

### MRI

Preoperative neuroimaging was performed using a standardized, high-resolution epilepsy protocol comprising T1-weighted, T2-weighted, inversion recovery (IR), short tau inversion recovery (STIR), and fluid-attenuated inversion recovery (FLAIR) sequences. To ensure accurate anatomical evaluation of mesial temporal structures and facilitate reproducible longitudinal assessments, image acquisition planes were strictly defined: axial sections were oriented parallel to the anterior commissure-posterior commissure (AC-PC) plane, while coronal slices were systematically angulated perpendicular to the long axis of the hippocampus.

### Hemispheric dominance evaluation

Preoperative hemispheric dominance was determined by applying the Edinburgh Handedness Inventory. This standardized questionnaire was utilized to quantify manual lateralization, providing a laterality quotient that served as a proxy for language dominance in our study cohort.

### Visual and neuropsychological evaluation

Preoperative assessment consisted primarily of clinical evaluation, serial scalp EEG recordings, and high-resolution MRI findings. Formal neuropsychological testing and standardized visual field perimetry were not routinely performed during the study period and were therefore unavailable for retrospective analysis.

### PET/SPECT and SEEG

Presurgical evaluation was based on clinical semiology, serial scalp EEG recordings, and high-resolution MRI findings. PET, SPECT, and invasive monitoring techniques such as SEEG were not routinely available during the study period and therefore were not systematically incorporated into the diagnostic workup.

### Outcomes

The primary outcome was defined as the achievement of complete freedom from disabling seizures (Engel Class I and ILAE Class 1) at 24 months post-radiosurgery. Secondary efficacy outcomes included the overall percentage reduction in seizure frequency from baseline, the proportion of responders (defined conventionally as patients achieving a ≥ 50% reduction in seizure frequency), and clinical outcome classifications using both Engel and ILAE scales at 12 and 24 months. Secondary safety outcomes encompassed the incidence and severity of acute and delayed adverse events, specifically focusing on visual field deficits (VFD), neurocognitive decline, and psychiatric complications.

### Therapeutic intervention

**Fig. 1:**
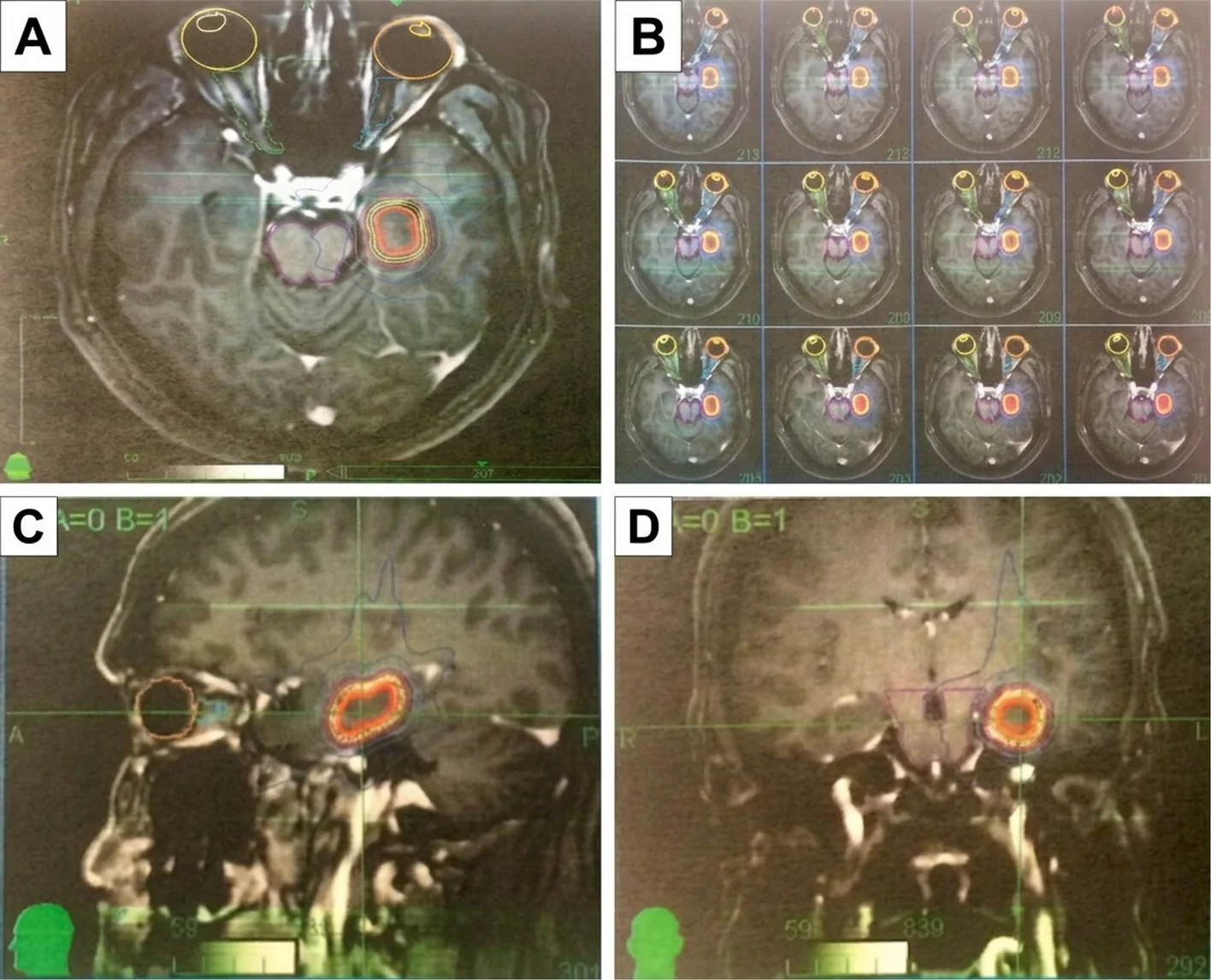
SRS Planning for MTLE. Representative LINAC-based radiosurgical plan targeting the hippocampal head and anterior body. **A** Axial view demonstrating the selective dose distribution encompassing the target volume while sparing adjacent temporal neocortical and brainstem structures. **B** Sequential axial slices showing the rostral-to-caudal extent of the treatment volume, illustrating high-conformity dose delivery. **C** Coronal view confirming target restriction to the mesial temporal region, with careful sparing of the optic chiasm and lateral temporal lobe. **D** Sagittal view confirming the limitation of the therapeutic volume to the anterior hippocampal segment, avoiding posterior hippocampal irradiation. Isodose lines are displayed in color-coded gradients, with the 50% isodose line highlighted as the prescription margin

Following diagnostic confirmation and obtaining written informed consent, patients were scheduled for an appointment at the Radiosurgery Department. A Novalis stereotactic frame was fixed under local anesthesia. For precise stereotactic alignment, planning CT scans were acquired utilizing contiguous 1-mm slice thickness in both non-contrast and contrast-enhanced phases. These images were co-registered with preoperative MRI using BrainLab software (Novalis system). Upon verification of image fusion, the target volume—encompassing the hippocampal head and anterior body—was precisely defined, and adjacent organs at risk (OARs) were delineated (Fig. 1).

Treatment planning was performed using a BrainLab Novalis LINAC with 6 MV photon energy, a micro-multileaf collimator, and the BrainScan Novalis Treatment Planning System (version 5.31), integrating stereotactic frame fixation, MRI, and contrast-enhanced CT data. The treatment geometry consisted of eight modulated dynamic arcs (gantry angles: 30°−150° and 210°−350°) and 30° couch rotations tailored to each patient´s anatomy.

The prescribed margin dose of 22 Gy was normalized to the 50% isodose line at the isocenter, ensuring ≥ 90% target coverage (V100 ≥ 90%) and a dose gradient < 10%. Dose constraints of OARs adhered to international standards: ≤ 8 Gy for the optic apparatus and cochlea, and < 10 Gy for the brainstem (volume < 1 mL). After plan validation and verification of the absence of collisions, a single prophylactic dose of 12 mg of dexamethasone was administered.

### Follow up

Hospitalization was not required, and all patients were discharged on the same day. A structured follow-up protocol was established, including weekly telephone monitoring and monthly outpatient visits. Patients and their families were instructed to maintain a detailed record of seizure and aura frequency. Comprehensive multimodal assessments—incorporating clinical, electroencephalographic, and neuroimaging evaluations—were conducted at 3, 6, 12, and 24 months post-intervention.

Clinical outcomes were classified at 12 and 24 months according to the criteria of the International League Against Epilepsy (ILAE), and at 24 months using the Engel classification. Anterior temporal lobectomy was established as a rescue surgical option for patients with treatment-refractory seizures or severe adverse events.

Seizure outcomes were evaluated using two standardized and widely accepted classification systems: the ILAE outcome classification and the Engel classification. Both instruments were applied at predefined follow-up intervals.

The ILAE outcome classification stratifies patients into six classes (1–6) based on postoperative seizure frequency, ranging from complete seizure freedom (Class 1) to no worthwhile improvement (Class 6). This system allows for a more granular assessment of seizure control, including the presence of auras and varying degrees of seizure reduction (see supplementary Table 1).

In parallel, the Engel classification categorizes outcomes into four classes (I–IV), where Class I indicates freedom from disabling seizures and Class IV reflects no significant improvement. Its widespread use facilitates comparison across historical and contemporary surgical and radiosurgical series (see supplementary Table 2).

During the 24-month follow-up, the baseline AEDs regimen remained strictly constant in four of the seven patients (57.1%). Pharmacological modifications were clinically necessitated in three patients (42.8%). Specifically, to manage the psychiatric and cognitive adverse events, one patient required the addition of targeted neuromodulators, including benzodiazepines and memantine. Two patients required the introduction of primidone and clonazepam, respectively. These required modifications highlight the clinical imperative to medically manage delayed radiation effects and persistent seizure activity in a subset of patients.

### Statistical analysis

Statistical analysis was performed using IBM SPSS Statistics for Windows, Version 26.0 (IBM Corp., Armonk, NY, USA). Continuous variables were expressed as medians and interquartile ranges (IQR). Due to the small sample size (n = 7) and non-normal distribution of the data (assessed by the Shapiro–Wilk test), non-parametric tests were used. The Friedman test was employed to evaluate longitudinal changes in seizure and aura frequency across time points (baseline, 6, 12, 18, and 24 months). Post hoc pairwise comparisons were performed using the Wilcoxon signed-rank test to identify significant differences between baseline and follow-up periods. Time-to-event outcomes, defined as the maintenance of a clinically meaningful seizure reduction (≥ 50% from baseline), were analyzed using Kaplan–Meier survival curves. A two-sided p-value < 0.05 was considered statistically significant.

### Ethical approval

The study was approved by the ethics committee of the Hospital de Especialidades, Centro Médico Nacional de Occidente (approval number R-2017–1301-57). Written informed consent was obtained from all patients for inclusion in the study and for the administration of radiotherapy.

## Results

A total of seven patients were included (3 females, 4 males). The median age was 40 years (IQR 32–57), with a median seizure duration of 31 years (IQR 22–35). Patients used a median of 3 AEDs, with valproate and levetiracetam being the most frequently prescribed. Regarding seizure type, all patients presented focal onset seizures with impaired awareness. Four patients reported auras, and four exhibited postictal periods with automatisms (Table 1).

**Table 1:** Baseline demographic characteristics

**Pt**	**Sex/Handedness**	**Age of Onset**	**Dominance**	**Pre-op MRI Findings**	**Pre-op EEG Foci**	**Clinical Cognitive Status**	**Target Vol (**cm^3^**)**	**Margin Dose (Gy)**	**Isodose Line (%)**
1	M/Right	3 years	Right	Right mesial temporal sclerosis	Right temporal high-voltage waves	Advanced cognitive decline	1.73	22	50
2	F/Right	37 years	Left	Left mesial temporal sclerosis	Left temporal high-voltage waves	No evident cognitive impairment	1.82	22	50
3	F/Right	12 months	Left	Left mesial temporal sclerosis	Left temporal high-voltage waves	No evident cognitive impairment	2.18	22	50
4	F/Right	29 years	Left	Left mesial temporal sclerosis	Left temporal high-voltage waves	No evident cognitive impairment	1.9	22	50
5	M/Right	7 years	Right	Right mesial temporal sclerosis	Right temporal high-voltage waves	No evident cognitive impairment	2.13	22	50
6	M/Right	1.5 years	Left	Left mesial temporal sclerosis	Left temporal high-voltage waves	No evident cognitive impairment	1.94	22	50
7	M/Right	22 years	Left	Left mesial temporal sclerosis	Left temporal high-voltage waves	No evident cognitive impairment	1.90	22	50

The overall median reduction in seizure frequency throughout the study was 75.6% (IQR 50% – 88.3%), although no patients became entirely seizure-free (Table 2).

**Table 2:** Baseline and postoperative seizure frequency and percentage reduction

Patient	Baseline (sz/mo)	6 Months (%)	12 Months (%)	18 Months (%)	24 Months (%)
1	15.0	3.1 (79.3)	3.2 (78.7)	2.8 (81.3)	2.2 (85.3)
2	10.0	7.0 (30.0)	5.4 (46.0)	5.2 (48.0)	8.0 (20.0)
3	8.0	1.1 (86.2)	0.5 (93.8)	0.6 (92.5)	0.6 (92.5)
4	12.0	1.1 (90.8)	0.8 (93.3)	1.7 (85.8)	3.5 (70.8)
5	12.0	1.1 (90.3)	1.0 (91.7)	1.4 (88.3)	1.4 (88.3)
6	8.0	2.6 (67.5)	3.1 (61.3)	4.3 (46.2)	4.0 (50.0)
7	25.0	3.6 (85.6)	7.5 (70.0)	7.2 (71.2)	6.1 (75.6)
Median	12.0	2.6 (85.6)	3.1 (78.7)	2.8 (81.3)	3.5 (75.6)
IQR	(8.0–15.0)	(67.5–90.3)	(61.3–93.3)	(48.0–88.3)	(50.0–88.3)

IQR, interquartile range; sz/mo, seizures per month. Data for individual patients are presented as absolute values with the corresponding percentage reduction from baseline in parentheses. Summary rows at the bottom represent the group median and IQR

The Friedman test revealed a statistically significant difference in seizure frequency across time points (χ^2^ = 14.62, p = 0.006). Post hoc analysis using the Wilcoxon signed-rank test demonstrated that these significant differences occurred between baseline and all follow-up periods. Notably, no significant differences were observed among the postoperative follow-up time points (Table 3), indicating a stable surgical outcome.

**Table 3:** Postoperative seizure frequency from baseline and all follow-up periods

Comparison	Z	*p-value*
Baseline – 6 Months	−2.37	0.018
Baseline – 12 Months	−2.36	0.018
Baseline – 18 Months	−2.36	0.018
Baseline – 24 Months	−2.36	0.018
6–12 Months	−0.16	0.866
6–18 Months	−0.33	0.735
6–24 Months	−1.52	0.128
12–18 Months	−0.33	0.735
12–24 Months	−0.84	0.398
18–24 Months	−0.16	0.866

Wilcoxon signed-rank test. *P*-values are unadjusted. Post-hoc comparisons were performed following a significant Friedman test

**Fig. 2:**
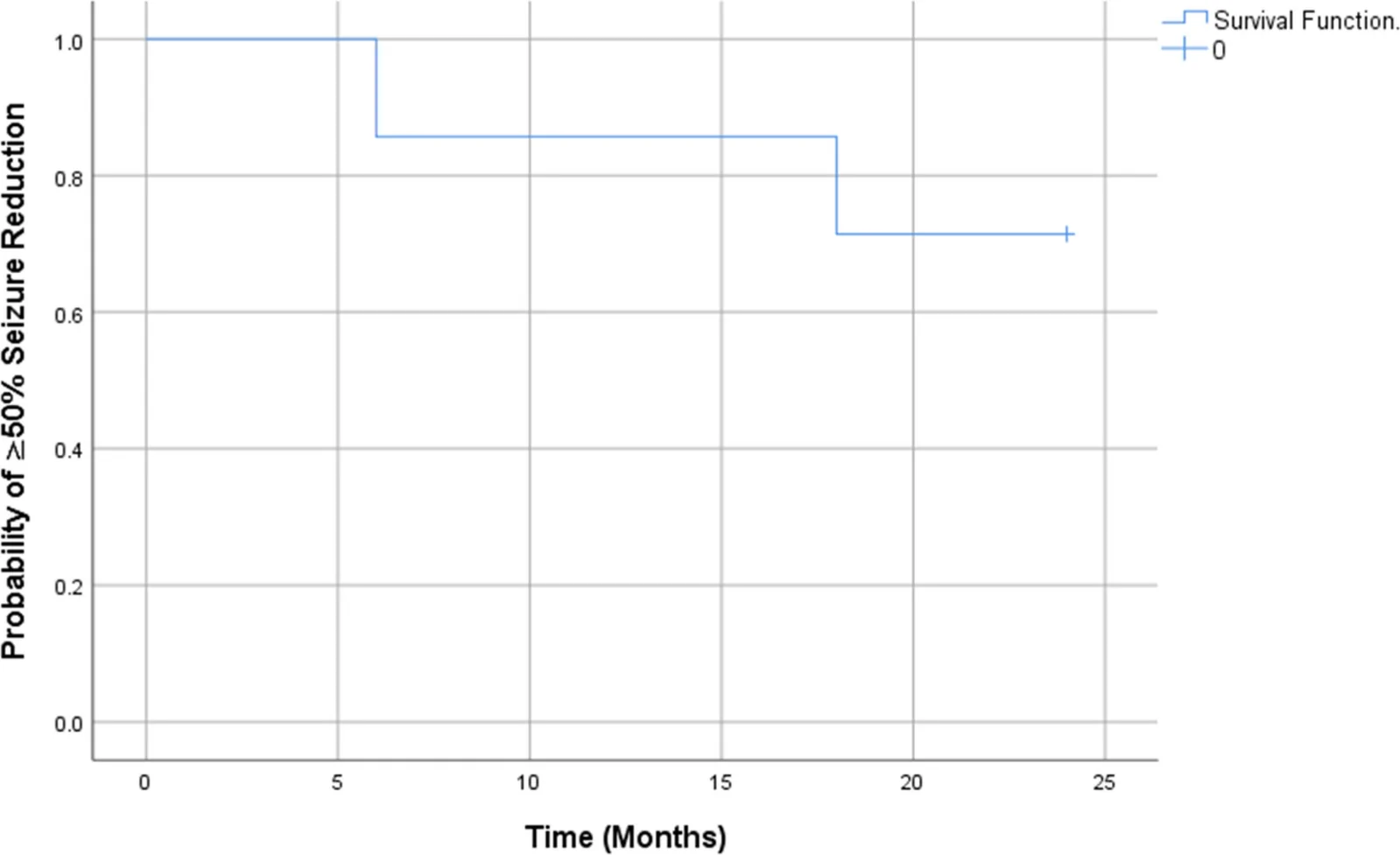
Kaplan–Meier curve of responder survival

Kaplan–Meier survival analysis demonstrated that the probability of maintaining a clinically meaningful seizure reduction (≥ 50% from baseline) was 85.7% at 6 months and 71.4% at 18 months. The survival curve remained stable thereafter through the 24-month follow-up, indicating a sustained therapeutic effect for the majority of the cohort. The median time to loss of response was not reached (Fig. 2).

The graph illustrates the probability of maintaining a clinically meaningful reduction in seizure frequency (≥ 50% from baseline) over 24 months.

Preoperatively, four patients presented with auras, while three were aura-free. Patients with preoperative auras, a clinical trend toward reduction was observed, with a median reduction of 60% at 24 months. Although a consistent reduction trend was noted, this did not reach statistical significance (Friedman χ^2^ = 8.632, p = 0.071). Notably, the three patients who were initially aura-free developed de novo auras during postoperative follow-up (Table 4).

**Table 4:** Aura frequency from baseline to 24 months

Patient	Baseline	6 Months (%)	12 Months (%)	18 Months (%)	24 Months (%)
1	40	2 (95)	1.5 (96.3)	2 (95)	1.5 (96.3)
2	30	16 (46.7)	26 (13.3)	23 (23.3)	21 (30)
3	8	1 (87.5)	0. 5 (93.8)	1 (87.5)	0.8 (90)
4	12	8 (33.3)	8.5 (29.2)	7.5 (37.5)	8.5 (29.2)
5	0	5 (NA)	1 (NA)	1 (NA)	1 (NA)
6	0	0 (NA)	0 (NA)	2 (NA)	2 (NA)
7	0	7.5 (NA)	11 (NA)	10.5 (NA)	11 (NA)
Median	21.0	5.0 (67.1)	5.0 (61.5)	4.8 (62.5)	5.0 (60.0)

Aura frequency is expressed as episodes per month. Patients 1–4 presented with preoperative auras, whereas patients 5–7 were aura-free at baseline. For the latter group, percentage reduction was considered not applicable (NA) due to the development of de novo symptoms

Follow-up outcomes at 12 and 24 months demonstrated overall stability in both the ILAE and Engel classifications. With the exception of one patient who exhibited a decline in seizure control, the classification scores remained unchanged, yielding no statistically significant difference between the two evaluation periods (Table 5).

**Table 5:** Postoperative ILAE and Engel for follow-up

Patient	ILAE at 12 Months	ILAE at 24 Months	Z	*p*-value
1	3	3	−1.000	0.317
2	5	5		
3	3	3		
4	3	4		
5	3	3		
6	4	4		
7	4	4		
Patient	Engel at 12 Months	Engel at 24 Months	Z	*p*-value
1	II	II	−1.000	0.317
2	IV	IV		
3	II	II		
4	II	III		
5	II	II		
6	III	III		
7	III	III		

Statistical comparison between 12-month and 24-month outcomes was performed using the Wilcoxon signed-rank test

Adverse events were documented during the 24-month follow-up period. VFD included bilateral hemianopsia in three patients (42.8%) and one case of recent memory loss in a patient with significant pre-existing cognitive impairment. These visual and cognitive deficits remain under multidisciplinary surveillance by the ophthalmology and neurology departments.

Psychotic episodes were documented in two patients, requiring specialized psychiatric intervention and continuous monitoring by the psychiatry department. Additionally, de novo auras were reported by three patients post-intervention. Systemic symptoms were limited to headaches in two patients, with no signs of papilledema, which resolved following a course of corticosteroids before the conclusion of the study.

## Discussion

Our study demonstrates that LINAC-based radiosurgery targeting the hippocampus and the anterior segment of the hippocampal body in patients with MTLE can achieve a clinically meaningful reduction in seizure frequency in selected patients with MTLE, although complete seizure freedom was not achieved and delayed adverse events were observed. These findings are consistent with previous reports describing delayed clinical effects attributable to both ischaemic necrosis and neuromodulation of epileptogenic tissue [10, 11].

Previous studies have defined a broader limbic target including the amygdalo-entorhinal complex. In contrast, our approach was restricted to the hippocampal head and anterior body, excluding extra-hippocampal limbic structures, and yielded a meaningful reduction in seizure frequency despite the more restricted target volume [12].

In comparison, the randomized ROSE trial demonstrated that while ATL achieves seizure-free rates of 70–78%, SRS administered at doses of 24 Gy to the 50% isodose targeting mesial structures achieved seizure-free rates of 52–60% at 24–36 months, albeit with less cognitive impairment [13–15]. Although our cohort did not yield any seizure-free patients, our results demonstrated a meaningful 75.6% reduction in seizure frequency, consistent with the seizure reducing effects reported in previous radiosurgical experiences using Gamma Knife (GK).

A recent systematic review of minimally invasive techniques (such as LITT, RFA, and SRS) demonstrated that ATL remains superior in terms of seizure control (60–80%) when compared to LITT and RFA (40–60%) and SRS (50–60%). However, this efficacy comes at the cost of a higher risk of permanent adverse effects. This evidence reinforces the role of radiosurgery as a valid alternative for patients with comorbidities, surgical contraindications, or a preference for minimally invasive procedures [16].

The latency of the clinical effect, usually between 9 and 24 months, appears to depend on progressive structural changes such as subnecrotic ischemic injury and alterations in the N-acetylaspartate/creatine (NAA/Cr) ratio observed by resonance spectroscopy [11, 17]. In our cohort, we observed a gradual decrease in seizure frequency that coincided with progressive neuroimaging changes, supporting the hypothesis of a delayed neuromodulation mechanism [7].

When evaluating the safety of radiosurgery, its adverse effects must be weighed against the established complications of open surgical resection. Specifically, the 90% incidence of VFD following open surgery for temporal lobe epilepsy is consistent with previous reports (9–100%), yet it surpasses the 62.5% rate found in an earlier SRS [18]. Moreover, major complications inherent to open surgery such as fatal outcomes, permanent motor deficits, major hemorrhage, and postoperative infections [12, 19] remain unobserved in radiosurgical applications. In spite of this, our current clinical findings reveal that LINAC therapy presents a safety profile that requires careful, nuanced interpretation. No severe clinically evident cognitive deterioration was observed, except in one patient with preexisting baseline impairment; nevertheless, our cohort experienced a substantial 42.8% rate of delayed VFD and a 28.6% incidence of severe psychiatric episodes requiring pharmacological management (one of whom had preexisting advanced cognitive decline). Rather than completely avoiding collateral damage, our data suggests that the visual pathways and perilesional limbic networks remain highly vulnerable to delayed radiation effects, underscoring the critical need for stricter dosimetric constraints.

Several studies show that GK radiosurgery for MTLE can cause side effects like headaches, nausea, depression, and vision problems, though severe complications are uncommon [12]. For instance, Régis et al. [14] reported new headaches in 14% of patients and VFD in 43%, while Barbaro et al. [12] noted these same issues in 70% and 50% of their patients, respectively. In our cohort, a similar profile was observed regarding only the presence of transient headaches; visual disturbances were frequently identified, at a percentage similar to previous studies. Additionally, a single case of cognitive deterioration was observed in a patient with pre-existing impairment.

Several radiosurgical series have described a transient increase in aura frequency during the first months following treatment. Quigg et al. and Rolston et al. reported that auras may temporarily worsen despite a reduction in disabling seizures, a phenomenon thought to reflect evolving neuromodulatory and radiobiological effects within epileptogenic networks. In particular, transient aura exacerbation has been correlated with the development of radiation-induced T2 hyperintensity and perilesional edema during the subacute post-radiosurgical period [19].

In our cohort, patients with pre-existing auras demonstrated an overall reduction in aura frequency over time; however, de novo auras developed in patients who were initially aura-free. Although the small sample size precludes definitive conclusions, these findings may support the hypothesis that radiosurgery initially alters seizure propagation pathways before achieving more complete network modulation. Similar observations have been reported in previous radiosurgical studies of mesial temporal lobe epilepsy.

Clinically, this phase may occur within the first year after treatment and has been associated with radiological changes such as T2 hyperintensity and edema at the target site. During this period, complex seizures tend to decrease, while auras may become more frequent [20].

As the radiosurgical lesion evolves over time, seizure frequency generally declines, consistent with the delayed therapeutic effect described in previous studies [15]. This may reflect progressive structural and functional changes within the epileptogenic network [21], although the precise mechanisms remain incompletely understood [19]. However, these observations should be interpreted with caution, and further studies incorporating functional connectivity analyses are needed to better characterize these network-level effects.

Among the strengths of this study are the precise dosimetric planning of the treated volume and the rigorous clinical follow-up, including periodic evaluations using standardized scales. However, several limitations must be acknowledged, such as the small sample size, the absence control group undergoing open surgery, and the duration of follow-up, which, although extending to 24 months, may be insufficient to fully assess the long-term durability of the therapeutic effect [16, 22]. Furthermore, the lack of formal preoperative neuropsychological batteries and automated visual perimetry limits our ability to precisely quantify the exact degree of cognitive and visual decline, relying instead on gross clinical symptomatic reporting. FDG-PET, SPECT, and SEEG were not routinely available and therefore were not incorporated into patient selection, potentially limiting the precision of epileptogenic zone localization.

Future research should focus on the design of controlled clinical trials directly comparing different minimally invasive techniques LINAC, GK and LITT, as well as the incorporation of structural and functional neuroimaging biomarkers to improve response prediction and enable individualized dosimetric planning [23].

## Conclusions

LINAC-based stereotactic radiosurgery targeting the hippocampal head and anterior body achieved a significant reduction in seizure frequency in patients with drug-resistant mesial temporal lobe epilepsy. However, complete seizure freedom was not achieved, and delayed adverse events, particularly visual field deficits and psychiatric complications, were observed.

The use of highly selective target volume restricted to the hippocampal head and anterior body, represents a feasible therapeutic approach for carefully selected patients who are not candidates for standard surgical resection or who lack access to alternative ablative technologies. Given the small sample size, the absence of advanced presurgical investigations, and the heterogeneous clinical characteristics of the cohort, these findings should be interpreted with caution.

## Supplementary Information

Supplementary Material 1.

## Data Availability

The clinical data used in this study are not publicly available due to institutional restrictions and patient confidentiality concerns, particularly given the risk of identification in a small sample size.

## Abbreviations

HS: Hippocampal sclerosis
ILAE: International League Against Epilepsy
MTLE: Mesial temporal lobe epilepsy
ATL: Anterior temporal lobectomy
LITT: Laser interstitial thermal therapy
RFA: Radiofrequency ablation
SRS: Stereotactic radiosurgery
LINAC: Linear accelerator- based radiosurgery
GK: Gamma Knife
IQR: Interquartile range
AEDs: Antiepileptic drugs
VFD: Visual Field Deficit
