# Supplementary material for: LINAC-based stereotactic radiosurgery targeting the hippocampus in mesial temporal lobe epilepsy: a single-center case series
Source: Chin Neurosurg J. 2026 Sep 8;12:27. doi: 10.1186/s41016-026-00446-6 (PMC13551764; doi:10.1186/s41016-026-00446-6)
Supplement: Supplementary file 1 [file 41016_2026_446_MOESM1_ESM.docx]

**Appendix**

**Supplementary Table 1: International League Against Epilepsy (ILAE) outcome classification.**

| Outcome classification | Definition |
| --- | --- |
| 1 | Completely seizure free; no auras |
| 2 | Only auras; no other seizures |
| 3 | One to three seizure days per year; ± auras |
| 4 | Four seizure days per year to 50% reduction of baseline seizure days; ± auras |
| 5 | Less than 50% reduction of baseline seizure days to 100% increase of baseline seizure days; ± auras |
| 6 | More than 100% increase of baseline seizure days; ± auras |

**Supplementary Table 2: Engel’s Classification of Postoperative Outcome**

| Class | Subclass | Definition |
| --- | --- | --- |
| I | A | Completely seizure free since surgery. |
|  | B | Nondisabling simple partial seizures only since surgery. |
|  | C | Some disabling seizures after surgery, but free of disabling seizures for at least 2 years. |
|  | D | Generalized convulsions with AED discontinuation only. |
| II | A | Initially free of disabling seizures but has rare seizures now. |
|  | B | Rare disabling seizures since surgery. |
|  | C | More than rare disabling seizures since surgery, but rare seizures for the last 2 years |
|  | D | Nocturnal seizures only |
| III | A | Worthwhile seizure reduction |
|  | B | Prolonged seizure-free intervals amounting to >50% of follow-up, but <2 years |
| IV | A | Significant seizure reduction |
|  | B | No appreciable change |
|  | C | Seizures worse |
